# Association of Tumor Necrosis Factor-α (-G308A) Polymorphism with risk of Upper Gastrointestinal Bleeding from Schistosomiasis in Pernambuco

**DOI:** 10.1590/0037-8682-0654-2021

**Published:** 2023-02-20

**Authors:** Bertandrelli Leopoldino de Lima, Elker Lene Santos de Lima, Maria Tereza Cartaxo Muniz, Ana Lúcia Coutinho Domingues, Paula Carolina Valença Silva

**Affiliations:** 1 Universidade Federal de Pernambuco, Centro Acadêmico de Vitória, Vitória de Santo Antão, PE, Brasil.; 2 Universidade de Pernambuco, Centro de Oncohematologia Pediátrica, Hospital Universitário Oswaldo Cruz, Laboratório de Biologia Molecular, Recife, PE, Brasil.; 3 Universidade de Pernambuco, Instituto de Ciências Biológicas, Recife, PE, Brasil.; 4 Universidade Federal de Pernambuco, Departamento de Medicina Clínica, Recife, PE, Brasil.

**Keywords:** Schistosoma mansoni, Tumor necrosis factor alpha, Genetic polymorphism, Gastrointestinal hemorrhage

## Abstract

**Background:**

We evaluated the association between polymorphisms in the tumor necrosis factor alpha (TNF*-α)* (-G308A) gene and *upper* gastrointestinal *bleeding* (UGIB) in schistosomiasis.

**Methods:**

This was a transverse study involving 294 Brazilian patients infected with *Schistosoma mansoni.*

**Results:**

The homozygous A/A genotype in TNF*-α* (-G308A) showed a risk association (prevalence ratio = 1.90, *p* = 0.008) with UGIB. There was no statistically significant difference in serum TNF-α levels between the clinical groups.

**Conclusions:**

The polymorphic TNF*-α* (-G308A) can be a risk factor for UGIB, in addition to being a potentially predictive factor for the severity of UGIB in schistosomiasis.

Schistosomiasis is a chronic parasitic infection that affects approximately 240 million people worldwide^1^. In Brazil, approximately 1.5 million people are presently infected by the hemoparasite *Schistosoma mansoni* (*S. mansoni*), especially in the Northeast region^2^ and mainly in the state of Pernambuco, which contains the highest number of deaths from this disease in the country^3^.

The pathological process of the disease occurs through an immune response to the parasite eggs deposited in the liver, causing a granulomatous inflammatory reaction^1^ ^,^ ^3^. This process causes periportal fibrosis (PPF), which leads to portal vein obstruction, followed by increased portal hypertension and the appearance of esophageal varices, which can rupture and cause *upper* gastrointestinal *bleeding* (UGIB)^1^ ^,^ ^2^ ^,^ ^4^. 

UGIB is a serious complication of the disease, especially in the advanced hepatosplenic form^4^. The probability of esophageal vessel rupture in schistosomiasis patients is 11-30%^5^, whereas the mortality caused by this complication is 10-20%^6^. Tumor necrosis factor-alpha (TNF-α) is an inflammatory cytokine that functions in the liver's regenerative process, forming hepatic granulomas and activating myofibroblasts^7^. This cytokine promotes the production of serum proteins by liver cells, such as fibrinogen and C-reactive protein, which causes an acute inflammatory reaction^6^.

The TNF*-α* gene is located in the human leukocyte antigen class III on chromosome 6p21.3^1^ ^.^ ^6^. Single nucleotide polymorphisms (SNP) are common in the promoter region of the TNF*-α* gene and may influence the function or expression of this protein^1^ ^,^ ^6^. Among these variations, one can find (-G308A) (rs1800629), in which there is a substitution of guanine (G) by adenine (A), creating two alleles (G, A) and three genotypes (AA, GG, and GA). In comparison with allele G, allele A is associated with increased levels of the TNF-α constituent^1^ ^,^ ^6^.

The association found between the polymorphisms and the increased level of the TNF-α constituent is a possible explanation of the evolutionary process of PPF^1^. When compared to genotype GG, genotype GA/AA represented a protective factor for regression of the pattern and degree of PPF^1^. Thus, it has been suggested that the TNF*-α* (-G308A) (rs1800629) polymorphism may be a prognostic factor for schistosomiatic PPF^1^. The impact of the expression of polymorphic variations in TNF*-α* and its effect on the severity of UGIB in patients with schistosomiasis needs to be better elucidated.

This study analyzed the association between TNF*-α* (-G308A) (rs1800629) and UGIB in *S. mansoni* endemic populations in the state of Pernambuco, Northeast Brazil.

This cross-sectional study was carried out from August 2020 to May 2021, with 294 individuals infected with *S. mansoni*, which determined the TNF*-α* (-G308A) polymorphism and serum concentrations. Diagnosis of the clinical forms of the disease was determined by the medical reports and physical examination of the patients, in addition to the evaluation of upper abdominal ultrasound to confirm the diagnosis and exclude other liver diseases. This ultrasound evaluation was performed only by the endoscopy operator of the Hospital das Clínicas/ Universidade Federal de Pernambuco (HC-UFPE) Unit, using equipment (Siemens Acuson S2000), with a convex transducer of 3.5-5.0 Mhz. To define the PPF pattern, the Niamey^7^ classification was used: A, no fibrosis; B, suspected fibrosis; C, light; D, moderate; E, advanced; and F, very advanced. Patients were also evaluated using upper digestive endoscopy to diagnose or exclude esophageal varices. 

These individuals were divided into two groups: Group 1 contained 108 individuals with the hepatosplenic (HE) form, advanced PPF (Pattern E or F), and Group 2 contained 186 individuals with the hepatointestinal (HI) form, with mild PPF (Pattern C) or without fibrosis (Pattern A), without UGIB. All participants were over 18 years of age and were treated at the Gastroenterology Clinic of the Hospital das Clínicas of the Federal University of Pernambuco (HC-UFPE), Recife, Brazil.

Patients with a history of other hepatic comorbidities, such as hepatitis B and C (examination performed through serology of antigens and antibodies), cirrhosis, hepatic steatosis, and PPF Pattern D (Niamey's classification) were evaluated using ultrasound, as other clinical forms of schistosomiasis were excluded. 

To determine GG, GA, and AA genotypes, biological samples were collected (blood) and the DNA was subjected to the polymerase chain technique for restriction fragment polymorphism analysis (PCR-RFLP), being isolated with the *Ncol* enzyme for single nucleotide polymorphism (SNP) detection in the promoter region TNF*-α* (rs1800629). In this region, the substitution of the nitrogenous base guanine (G) by adenine (A) occurs at position -308 of gene^1^.

An immunoenzymatic assay was performed using a commercial ELISA kit (Biosource, Invitrogen Corporation, Carlsbad, CA, USA) following the manufacturer's instructions to measure the serum levels of TNF-α. Results are expressed in pg/mL, based on standard curves (sensitivity <1.7 pg/mL). The median cut-off point was the median 32pg/mL and 148 individuals were evaluated at this stage.

Crude prevalence ratio (PR) and 95% confidence intervals (95% CI) were estimated. The association between the TNF*-α* (-G308A) polymorphism and UGIB was evaluated with Fisher’s exact test or Chi-square test at a significance level of 0.05. Epi-Info software version 3.5.5 (CDC, Atlanta, GA, USA) was used, and the result was considered significant when p< 0.05.

This study was approved by the Ethics and Research Committee of the Center for Health Sciences, UFPE, under protocols 113.199 and 03161512.6.0000.5208.

The mean age of the participants was 48 years (standard deviation, 14 years). There was no statistically significant difference in the sex distribution (*p*= 0.192). There was no evidence of an association between serum TNF-α levels and the clinical groups (PR=1.472, 95% CI=0.8199 - 2.6428, *p*=0.2221) (Table 1).

**TABLE 1:** Analysis of the median serum levels of TNF-α in patients with schistosomiasis between the clinical groups, Pernambuco, Brazil.

TNF-α and UGIB by Dosage							
	Yes (n=72)		No (n=76)				
Characteristics	N	%	N	%	PR	95% CI	***p*-value**
Dosage TNF-α*							
<32 pg/mL	64	89	61	80	1.472	[0.8199 - 2.6428]	0.2221
>32 pg/mL	8	11	15	20			
**Total**	**72**	**100**	**76**	**100**			

*148 patients were evaluated for serum TNF-α dosage; **TNF-α:** tumor necrosis factor-alpha; UGIB: upper gastrointestinal bleeding; **PR:** prevalence ratio; **CI:** confidence interval.

Genotype AA showed a risk association (PR=1.90, 95% CI=1.322-2.757, *p*=0.008) for UGIB, when compared with genotypes GG and GA (Table 2).

**TABLE 2:** Bivariate analysis of associations between polymorphic *TNF-α* (-G308A) and UGIB, in patients with schistosomiasis in the state of Pernambuco, Brazil.

	**Genotypes for *TNF-α* -308 and UGIB**							
	Yes (n=108)		No (n=186)				
Genotypes	n	%	n	%	PR	95% CI	***p*-value**
**TNF*-α* (-308)**							
GG	55	51	106	57		(Reference)	
GA	38	35	72	39	1.01	[0.723 - 1.413]	1
AA	15	14	8	4	1.90	[1.322 - 2.757]	0.008
**Total**	**108**	**100**	**186**	**100**			

TNF-α: tumor necrosis fator-alpha, UGIB: Upper gastrointestinal bleeding; PR: prevalence ratio; CI: confidence interval.

This study investigated the impact of the TNF*-α* (-G308A) polymorphism on UGIB. This is the first study to analyze the influence of genetic factors on UGIB in a group of patients with schistosomiasis in the state of Pernambuco, Northeastern Brazil, a region with a high mortality rate in the country^5^.

This study involving Brazilian patients infected with *S. mansoni* showed that patients with genotype AA had a higher risk of UGIB than those with genotype GG. However, there was no association between serum TNF-α levels in the clinical groups of UGIB.

UGIB is caused by chronic schistosomiasis. UGIB can occur in up to 80% of patients with PPF, with a mortality rate of up to 30% for recurrent episodes. However, not all patients with advanced fibrosis develop esophageal varices, and not all patients with esophageal varices will have UGIB. The frequency of UGIB in Brazil is lower than that in Africa^8^. In addition, mainly in the presence of early grade fibrosis and in young-age patients, the effect on portal hypertension may even precede that on fibrosis and occur in patients with different grades of liver fibrosis^7^. Clinical factors such as esophageal varices and PPF are associated with a higher probability of having two or more cases of UGIB during life^5^ ^,^ ^9^. Opio et al. (2016)^9^ investigated in a cross-sectional study, Opio et al. investigated the profile of upper gastrointestinal bleeding episodes in 107 patients with schistosomiasis from Africa; 94% had severe PPF, and 80% had esophageal varices. A total of 323 occurrences of UGIB were identified throughout the lives of these individuals, with 57% having more than two episodes.

The association between the TNF*-α* (-G308A) polymorphism and PPF has already been studied^1^. Oliveira et al. (2015)^1^, through a cohort study, evaluated 124 Brazilians infected with *S. mansoni* after specific treatment and found a protective association between the genotypes GA/AA and the regression of PPF compared to the genotype GG. There was no association between serum TNF-α levels and the exposed groups; however, it is believed that the TNF*-α* (-G308A) polymorphism can prevent regression of the pattern and grade of PPF, which is a potential prognostic factor in relation to schistosomiasis PPF.

Silva et al. (2017)^4^ carried out a cross-sectional study of 256 Brazilians with schistosomiasis and different patterns of PPF and found that the AA genotype (-308) was associated with an increased risk of advanced PPF in this population. Serum TNF-α levels were higher in patients with moderate-to-advanced PPF than in those with a mild pattern. Therefore, the AA genotype is considered a risk factor for the severity of advanced PPF in this population.

In humans, although high TNF-α levels are associated with severe hepatic fibrosis^1^ ^,^ ^4^, it is still unclear which cytokines are involved in the progression of schistosomiasis pathology. 

Some studies have found no association between this polymorphism and the severity of PPF and hepatic fibrosis^10^ ^,^ ^11^. Moukoko et al. (2003)^10^ observed in a Sudanese population that high levels of TNF-α were associated with a higher risk of PPF but found no association between the TNF*-α* (-G308A) polymorphism and PPF. These authors did not exclude the possibility that this polymorphism influenced the development of PPF. 

Kusumoto et al. (2006)^11^ evaluated 460 hepatitis C vírus (HCV)-positive patients and 63 controls in Japan and found no significant association between this polymorphism and hepatic fibrosis. These authors concluded that these results may have been influenced by ethnic variations and that the TNF*-α* (-G308A) polymorphism can be associated with hepatic fibrosis. 

The influence of this polymorphism on the expression of TNF*-α*. Some studies have shown that, in comparison with allele G, allele A was associated with increased levels of TNF-α, while others failed to associate the presence of the A allele with different levels of TNF-α production^12^. These results may validate the hypothesis of this study, which showed that the tested genotype AA may play an important predictive role for PPF and, consequently, for UGIB in schistosomiasis in the Brazilian population.

In the current study, although there was no difference in serum TNF-α levels between the clinical groups of UGIB, possibly individuals with the genotype AA maintained a residual production of TNF-α after the specific treatment, contributing to less reabsorption of fibrosis and consequently causing complications by UGIB in this group.

The absence of a correlation between TNF-α levels and disease severity, represented by the degree of liver fibrosis, is also shown. There are controversial reports regarding the influence of TNF-α on schistosomiasis pathogenesis. Franco et al. (2021)^13^ evaluated 69 patients with schistosomiasis recruited from an endemic area for schistosomiasis in the north of the State of Sergipe, Brazil. There were no significant correlations between TNF-α levels and advanced degree of fibrosis. Therefore, owing to the small sample size, further studies are necessary to rule out the influence of TNF-α on schistosomiasis pathogenesis.

Another study did not differ between TNF-α levels in obese individuals who underwent bariatric surgery with or without liver fibrosis. The authors reported some limitations to be considered, including a small population of patients, which is mainly due to the high costs of the trials used in this study. In addition, there was no control group, and all individuals evaluated presented some degree of nonalcoholic fatty liver disease, which may limit a better elucidation of the findings and additional extrapolation of these results^14^. In addition, the effect of TNF-α on acute liver injury is unclear, and it has been reported that TNF-α plays a dual role in acute liver injury^15^. 

Considering the limitation of sample size in this study, as well as possible ethnic variations in the population, further studies with larger samples and in other populations are recommended to better analyze these genotypes and their respective doses of TNF-α serum, to better assess whether there is a connection between the TNF*-α* (-G308A) polymorphism and the expression of TNF*-α* and UGIB intensity in schistosomiasis. 

In conclusion, these results suggest that the TNF*-α* (-G308A) polymorphism can be a risk factor for UGIB in the Brazilian population, and therefore, can potentially be a predictive factor for the severity of UGIB resulting from PPF in schistosomiasis.
